# Identification of anticancer drug target genes using an outside competitive dynamics model on cancer signaling networks

**DOI:** 10.1038/s41598-021-93336-z

**Published:** 2021-07-08

**Authors:** Tien-Dzung Tran, Duc-Tinh Pham

**Affiliations:** 1 Complex Systems and Bioinformatics Lab, Faculty of Information and Communication Technology, Hanoi University of Industry, Bac Tu Liem District, 298 Cau Dien street, Hanoi, Vietnam; 2 Department of Software Engineering, Faculty of Information and Communication Technology, Hanoi University of Industry, Bac Tu Liem District, 298 Cau Dien street, Hanoi, Vietnam; 3 Graduate University of Science and Technology, Vietnam Academy of Science and Technology, Hanoi, Vietnam

**Keywords:** Cancer, Cancer therapy, Targeted therapies, Dynamical systems, Computer science, Systems biology, Systems analysis, Target identification

## Abstract

Each cancer type has its own molecular signaling network. Analyzing the dynamics of molecular signaling networks can provide useful information for identifying drug target genes. In the present study, we consider an on-network dynamics model—the outside competitive dynamics model—wherein an inside leader and an opponent competitor outside the system have fixed and different states, and each normal agent adjusts its state according to a distributed consensus protocol. If any normal agent links to the external competitor, the state of each normal agent will converge to a stable value, indicating support to the leader against the impact of the competitor. We determined the total support of normal agents to each leader in various networks and observed that the total support correlates with hierarchical closeness, which identifies biomarker genes in a cancer signaling network. Of note, by experimenting on 17 cancer signaling networks from the KEGG database, we observed that 82% of the genes among the top 3 agents with the highest total support are anticancer drug target genes. This result outperforms those of four previous prediction methods of common cancer drug targets. Our study indicates that driver agents with high support from the other agents against the impact of the external opponent agent are most likely to be anticancer drug target genes.

## Introduction

Drugs bind to their target proteins/genes, which regulate downstream effectors and ultimately perturb the transcriptome of a cancer cell. Identification of novel drug target genes is a significant challenge in anticancer drug development^1–3^. In recent studies, the phenotypic effects and chemical structures of drugs have been used to infer drug–gene pairs. The phenotypic effect-based approaches exploit the various phenotypic responses, such as expression profiles and side effects, to external anticancer compounds^4–7^. On the assumption that structurally similar drugs tend to bind to similar genes, chemical structure-based approaches have been implemented and have shown promising results^8–10^. Although substantial progress has been made in this field, numerous challenges remain to be addressed. In phenotypic effect-based approaches, the drugs affecting different targets in the same pathway or in the same biological process may cause similar drug responses; in addition, gene expression patterns cannot distinguish target genes from downstream-regulated genes. Moreover, reportedly, the gene expression of drug targets is typically insignificantly affected by drug perturbation. Therefore, autonomous gene expression changes following drug treatment are insufficient to identify drug targets^11^. Chemical structure-based approaches often rely on a few proteins^12,13^, such as those with known interacting drugs^14,15^ or with known three dimensional (3D) structures^8,10^. These approaches are insufficient for most proteins without such prior information.

In the past decade, anticancer drug target prediction has gained more interest with the availability of molecular biological network data, such as metabolic networks^16,17^, protein–protein interaction networks, gene regulatory networks^18^, heterogeneous network^19–22^, and cancer signaling networks^23^. Description and analysis of the network data provide a systematic understanding of drug action and disease complexity as well as help improve the efficiency of anticancer drug design^24^. Therefore, network-based methods have been developed to analyze the structure and dynamics of molecular biological networks for improving several stages of drug discovery, particularly to predict drug target genes and designate new therapies^25–29^. Among cancer-related networks, cancer signaling networks are a heterogeneous network type and provide the most informative data for dynamics analysis because they contain both directed and undirected interaction types, rather than containing only one interaction type as observed with the other network types^30,31^. Additionally, if no additional data (such as gene expression data) are integrated into the analysis, computation on these networks often yield more precise prediction results compared with that on the others^23^. Structural analysis revealed that cancer biomarker genes, which lead to cancer via mutations, often reside at high hierarchical closeness positions in the innermost core of the cancer signaling networks^25^. Particularly, in dynamics analysis, the backbone driver nodes found in cancer signaling networks can drive the network into a cancer phenotype as well as steer it into a healthy phenotype. This means that backbone driver genes could be cancer biomarkers as well as cancer therapeutic targets^32^. Unfortunately, determining optimal driver nodes for drug targets in actual biological networks remains a challenge^33^, and a dynamic model for anticancer drug target identification requires further studies.

Recently, Zhao et al.^34^ introduced a dynamic model that involved competition among two competitors for obtaining a maximum number of votes from other agents in a social network, where the two competitors within the same network have fixed and different states and each normal agent adjusts its state according to a distributed consensus protocol. The model can predict the bias of each normal agent and thus predict the competitor that will win. They found that the competition result completely depended on the network structure and positions of competitors in the network. Furthermore, it was observed the competitor with higher PageRank in a directed network or higher Katz Centrality in an undirected network has the highest likelihood to be the winner. Although these findings are extremely interesting, the research did not consider the case that one competitor is inside the network whereas the other is outside. This case is an extremely common phenomenon that often occurs in the field of social network and in the field of molecular biological network. In a social network, leaders inside the network often must counter the influence of competitors outside the system^35,36^. Similarly, in a molecular biological network, an environmental agent, such as UV radiation, drugs, chemicals, and viruses, can be considered the external competitor that causes perturbation against the signals of driver agents within the network^37^. Therefore, the external competition between two competitors can be considered a competition between an internal leader and an external opponent competitor in the social network or between a driver agent and an environmental agent in the cancer signaling network for obtaining maximum support from other agents in the network.

Here, we propose a dynamical network model called an outside competitive dynamics model in which an inside leader (driver agent, e.g., a drug target gene) and an opponent competitor outside the system (environmental agent, e.g., a drug) have fixed and different states. If any normal agent links to the external environmental competitor, the state of each normal agent will converge to a stable value, indicating support to (or impact from) the leader against the impact of the competitor. We showed an illustrative example of the working of the model in a disease network, which is formed by the integration of pathways related to a human disease such as a cancer, and the influence of adjacency weights on the outside competition results. We calculated the total support of normal agents to each leader in various networks (i.e., 17 actual and 100 random networks) and observed that the total support positively correlates with hierarchical closeness, the highest rankings of which were used to identify biomarker genes, which have been reported as therapeutic cancer targets in a cancer signaling network^32^. To reinforce the result observed, we gave an illustrative example to show that hierarchical closeness outperforms other popular centralities in the prediction for total support of a node. Interestingly, by experimenting on 17 cancer signaling networks downloaded from the Kyoto Encyclopedia of Genes and Genomes (KEGG), we found that 82% of the genes among the top 3 agents with the highest total support are anticancer drug target genes. This result implies that genes with high support from the other genes against the impact of the external opponent agent are most likely to be anticancer drug target genes in a cancer signaling network. In other words, the top three agents with the highest total support may play driver nodes in a complex network. Finally, we used prediction results on common cancer drug targets of four previous network-based methods to validate our results. As a result, our top 1 prediction shared the highest consistency with the previous predictions. Overall, the outside competitive dynamics model contributes to the identification of both anticancer drug targets and driver agents in the cancer signaling network.

## Material and methods

### Overview of the process for identifying anticancer drug target genes

**Figure 1:**
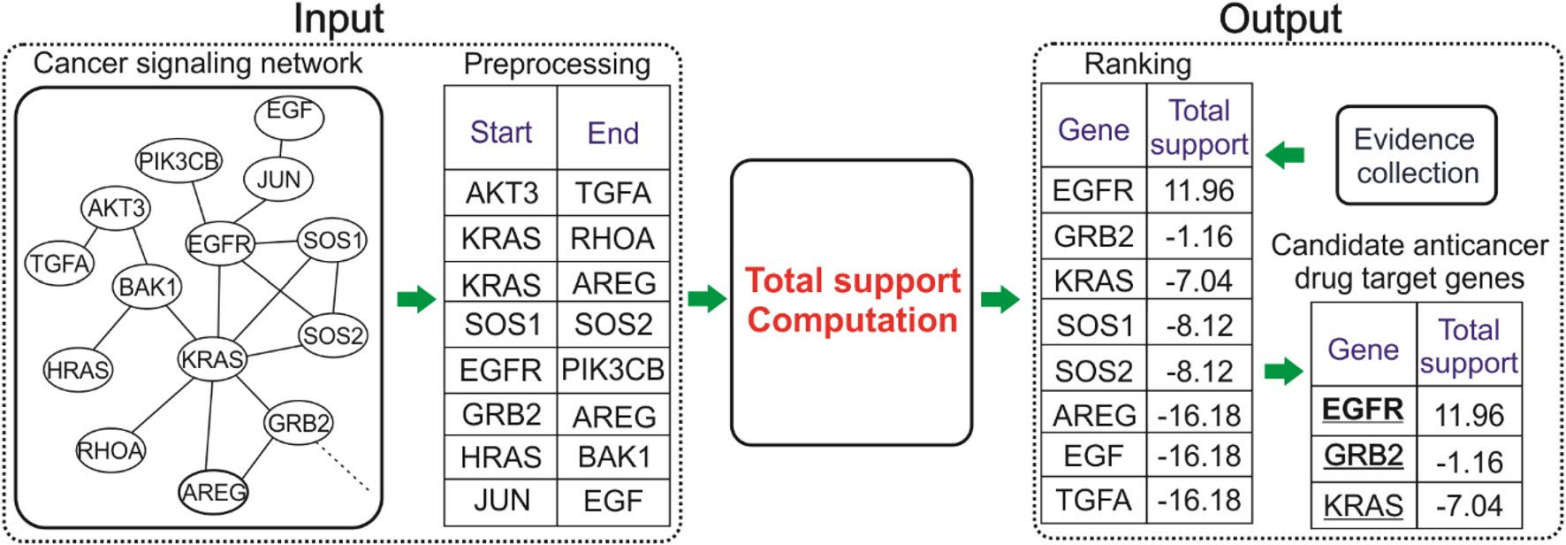
Process to identify anticancer drug target genes using an outside competitive dynamics model. From an input network, the process uses an algorithm based on the proposed model to compute the total support score of nodes in the network to each node for selecting the top three highest-ranking nodes, which are considered drug target genes as the output.

The process to identify anticancer drug target genes using the outside competitive dynamics model is presented in Fig. 1. In the viewpoint of our study, a drug target gene should be a driver node of a disease network^32^. Therefore, this process factually detects the driver nodes of a disease network. First, a disease network is used as the input data of the process. In this study, each of 17 cancer signaling networks downloaded from the KEGG database was used as the input data in turn. Second, the network is pre-processed by replacing each group node (if any) with single nodes interacting with each other to form a heterogeneous network that includes two types of undirected and directed links. Third, the outside competitive model is applied to the network to identify driver nodes. To deploy the model on a network, each normal node is randomly assigned a state between -1 and 1, but the inside leader node, e.g., a driver gene, and the outside opponent competitor, e.g., a drug, that links to the normal node are fixed by opposition states. A random walk process is then simulated to determine whole network steady states, where can thereby compute the total steady-state, called total support, of every normal node to the leader. The algorithm of total support computation considers each node as a trial leader in competition with the outside competitor, and the trial leader node with the highest total support is eventually selected as the driver node. Because identification of driver nodes may be approximate due to noise from network construction, we selected driver nodes from the top three highest total support, and they are also candidate anticancer drug target genes. Finally, these candidate anticancer drug target genes are matched with available evidence to identify real drug target genes and promising ones. Besides cancer, this process can be applied widely for cardiovascular, neurodegenerative, or other diseases with pathways available in open databases such as KEGG and BioCarta.

### Cancer signaling networks

We downloaded 17 cancer pathways from the KEGG database (www.genome.jp/kegg)^38–40^ for conducting network analysis. Other pathway databases, such as BioCyc (biocyc.org)^41^, Reactome (www. reactome. org)^42^, and BioGRID (thebiogrid.org)^43^, were not considered for analysis because they do not include pathways corresponding to a specific cancer site. Each cancer pathway was represented by a heterogeneous network, wherein a node and a link correspond to a protein and a protein–protein interaction, respectively. In the network, undirected links represent protein–protein interactions including binding/association and dissociation whereas directed links represent activation, inhibition, expression, indirect effect, interaction via compound, missing interaction, and phosphorylation. Cytoscape plugin KEGGParser^44^ was used to correct the pathways after they were downloaded from KEGG pathway database because the original KGML (KEGG Markup Language) files were inconsistent to the static pathway map image. In addition, an interaction from a protein *A* to a group of proteins {*B*_1_, *B*_2_,…, *B*_k_} in the original KEGG pathways was transformed into *k* different interactions of *A* → *B*_1_, *A* → *B*_2_,…, and *A* → *B*_*k*_ in the signaling network^45^.

### Computation of centrality measures

Considering a heterogeneous network *G*(*V*, *E*)*,* we briefly introduce two well-known structural centrality measures as follows.

Closeness centrality: The closeness centrality^46^ of a node *u* is defined as follows:

1 $$C_{{clo}} (u) = \frac{1}{{\sum\nolimits_{{v \in V\backslash {{\{ u\} }}}} {d(u,v)} }},$$

where *d*(*u,v*) is the shortest distance from node *u* to node *ν*. This measure has previously been used to prioritize disease genes in a protein–protein interaction network^47,48^. However, the definition of *C*_*clo*_(*u*) is not proper in cases where there is a node *v* that is not reachable from *u* because *C*_*clo*_(*u*) eventually becomes zero. Therefore, we used another version of closeness^49^ as follows:

2 $$C_{{clo - v}} (u) = \frac{1}{{\left| V \right| - 1}}\sum\limits_{{v \in V\backslash {{\{ u\} }}}} {\frac{1}{{d(u,v)}}}$$

Hierarchical closeness: Hierarchical closeness^29^ of a node u is *C*_*hc*_(*u*) and is determined by combining reachability and closeness measures as follows:

3 $$C_{{hc}} (u) = N_{R} (u) + C_{{clo - u}} (u)$$

where *N*_*R*_(*u*) ∈ [0*,|V|*−1] is the reachability of a node *u* defined by *N*_*R*_(*u*) =*|*{*v ∈ V|*∃ a path from *u* to *v*}|. Hierarchical closeness measure was successfully used to identify biomarker genes^25^ and disease genes^29^ on heterogeneous biological networks such as cancer signaling networks. In the present study, we used the finding regarding hierarchical closeness as evidence to support our results.

## Results

### A dynamic model for external competition

We consider a disease network *G*(*V,E*) with *N* agents and *M* links. The agent (node/gene) set is denoted as *V* = {1, 2,…, N}, and the topology of the network is described by an adjacency matrix *S* = (s_*uv*_)_*NxN*_. If agent *u* is directly influenced by agent *ν*, then there is a link from agent *u* to agent *ν* and *S*_*uv*_ ∈ (0, 1] indicating the weight of the link; otherwise, *S*_*uv*_ = *0*. For example, *S*_*uv*_ ∈ {0, 1} indicating the existence of links in biological networks^50^, and *S*_*uv*_ ∈ (0, 1] indicating the strength of ties in weighted biological network^51^. We assumed that node *α* ∈ *V* is a leader agent (a driver agent, e.g., a drug target gene) and node *β* ∉ *V* is an outside opponent competitor (an environmental agent, e.g., a drug), where the state of the leader agent and the opponent agent have fixed and different states as follows:

4 $$x_{\alpha } \left( t \right) = + 1,\forall t \ge 0{\text{; }}x_{\beta } \left( t \right) = - 1,{\text{ }}\forall t \ge 0$$

There is an unknown link that may connect from *β* to any node in the network for causing perturbation against α. Biologically, this link represents the transmission of the effect of drug *β* on any node against the impact of the driver agent *α*. Therefore, it was assumed that an undirected link was temporarily added between node *β* and any node *γ* ∈ *V*, whenever *γ* adjusts its state. Every agent (called a normal node) denoted as *u* ∈ *V*/{α, *β*} has a random initial state and updates its state as follows:

5 $$x_{u} \left( {t + 1} \right) = x_{u} \left( t \right) + \varepsilon \mathop \sum \limits_{v}^{{N_{u} }} S_{{uv}} \left( {x_{v} \left( t \right) - x_{u} \left( t \right)} \right){\text{ }}$$

where *x*_*u*_(*t*) is the state of agent *u* at time *t*; the parameter ${\text{0 < }}\varepsilon {\text{ < W}}_{{max}}^{{ - 1}}$ captures the level of neighbors’ influence, with *W*_*max*_ being the largest total weights of out-links of nodes in the network; and *N*_*u*_ = {*v* ∈ *V|S*_*uv*_ ∈ (0, 1]} is the set of neighboring nodes of node *u* that can directly influence node *u*. Equation (5) represents distributed consensus protocols proposed in the classical model of DeGroot^52^. The existence of competitors in the network disallows global consensus. Equation (5) biologically implies that expression state of a gene *u* for next period denoted by *y*_*u*_(*t* + *1*) ∈ [0, 1] can be predicted by its current state *y*_*u*_(*t*) plus an error term, where *y*_*u*_(*t*) = (*x*_*u*_(*t*) + 1)/2, *x*_*u*_(*t*) ∈ [-1, 1]. With *t → ∞,* the state of each normal node *u* converges to a steady value $\bar{x}_{u}$, which is a convex combination of the opponent states and independent of the initial states of nodes. The sign of the steady state of each normal node: $\bar{x}_{u}$ > 0 ($\bar{x}_{u}$ < 0) implies that node *u* will finally support or impacted by leader node α (opponent node *β*), and $\left| {\bar{x}_{u} } \right|~$ corresponds to the degree of support or impact. $\bar{x}_{u}$ = 0 if node *u* is a neutral node. In other words, this result means that if the random walk process^53^ in Eq. (5) converges, it will determine whether the expression state of each normal gene is eventually more affected by drug *β* or by driver agent *α*.

#### **Theorem 1**

*Given is a set* *X*_*norm*_* ∈ R*^*N*−2^, *which represents the state vector of all normal nodes in the network G(V,E) above.*

6 $$\mathop {X_{{norm}} }\limits_{{t \to \infty }} \left( t \right)\to\bar{X} \triangleq (\bar{D} - \bar{S})^{{ - 1}} \left[ {c_{\alpha } c_{\beta } } \right]\left[ {\begin{array}{*{20}c} { + 1} \\ { - 1} \\ \end{array} } \right],$$

where $\bar{D},~\bar{S},~\left[ {c_{\alpha } ~~c_{\beta } } \right]~$ can all be derived from the network adjacency matrix *S*. If *x*_*u*_(0) ∈ [− 1, + 1]*,* ∀*u* ∈ V/{α, *β*}*,* then *x*_*u*_(*t*) ∈ [− 1, + 1]*,* ∀*t* > 0.

*Proof of Theorem 1* We can rewrite Eq. (4 + 5) in the following matrix form:

7 $$X(t + 1) = (I_{N} - \varepsilon H.L)X(t) = TX(t)$$

where *I*_*N*_ is an identity matrix; *L* = *D-S* is the Laplacian matrix, *D* is the diagonal matrix where *D*_*uu*_ = $\mathop \sum \limits_{v} S_{{uv}}$; *H* is an indicative diagonal matrix with *H*(*i*, *i*) = *0* if agent *i* is a competitor and *H*(*i*, *i*) = 1 otherwise. Indeed, the sum of each row of matrix *T* equals 1 because the sum of each row of matrix *L* always equals 0; therefore, the sum of each row of the matrix *ε.H.L* equals 0. For convenience, we reordered the agents to ensure that the two competitors come last. Accordingly, we have the following:

8 $$D = \left[ {\begin{array}{*{20}c} {\overline{D} } & 0 & 0 \\ 0 & {d_{\alpha } } & 0 \\ 0 & 0 & {d_{\beta } } \\ \end{array} } \right]{\text{ }}and{\text{ S = }}\left[ {\begin{array}{*{20}c} {\overline{S} } & {c_{\alpha } } & {c_{\beta } } \\ {r_{\alpha } } & 0 & * \\ {r_{\beta } } & * & 0 \\ \end{array} } \right]$$

where *d*_α_ and *d*_*β*_ denote the total weights of out-links of competitor α and *β*, respectively; vectors *c*_α_*, c*_*β*_*, r*_α_, and *r*_*β*_ contain the corresponding elements in the reordered adjacency matrix. Hence, Eq. (7) can be rewritten as follows:

9 $$\left[ {\begin{array}{*{20}c} {X_{{norm}} (t + 1)} \\ {x_{\alpha } (t + 1)} \\ {x_{\beta } (t + 1)} \\ \end{array} } \right] = \left[ {\begin{array}{*{20}c} Q & {\begin{array}{*{20}c} {} & B \\ \end{array} } & {} \\ 0 & 1 & 0 \\ 0 & 0 & 1 \\ \end{array} } \right]\left[ {\begin{array}{*{20}c} {X_{{norm}} (t)} \\ {x_{\alpha } (t)} \\ {x_{\beta } (t)} \\ \end{array} } \right],$$

where $X_{{norm}} \in R^{{N - 2}}$ represents the state vector of all normal agents; $Q = I_{{N - 2}} - \varepsilon \left( {\bar{D} - \bar{S}} \right){\text{ and B = }}\varepsilon \left[ {c_{\alpha } {\text{ c}}_{\beta } } \right].{\text{Thus,}}$

10 $$X_{{norm}} (t) = QX_{{norm}} (t - 1) + B\left[ {\begin{array}{*{20}c} {x_{\alpha } (t - 1)} \\ {x_{\beta } (t - 1)} \\ \end{array} } \right] = Q^{t} X_{{norm}} (0) + \sum\limits_{{u = 0}}^{{t - 1}} {Q^{u} B\left[ {\begin{array}{*{20}c} {x_{\alpha } (0)} \\ {x_{\beta } (0)} \\ \end{array} } \right]} {\text{ }}$$

If each normal agent has a path connecting to at least one competitor, then $\bar{D} - \bar{S} \in R^{{N - 2}} {\text{~}}$ is invertible. Because ${\text{0 < }}\varepsilon {\text{ < }}W_{{max}}^{{ - 1{\text{~}}}}$, from the Gersgorin disk theorem, it can be demonstrated that the spectral radius of *Q* is less than 1. Thus, as $t \to \infty$, we have the following:

11 $$X_{{norm}} \left( t \right){\to}(I_{{N - 2}} - Q)^{{ - 1}} B\left[ {\begin{array}{*{20}c} {x_{\alpha } \left( 0 \right)} \\ {x_{\beta } \left( 0 \right)} \\ \end{array} } \right] = (I_{{N - 2}} - I_{{N - 2}} + \bar{D} - \bar{S})^{{ - 1}} \left[ {c_{\alpha } {\text{ }}c_{\beta } } \right]\left[ {\begin{array}{*{20}c} {x_{\alpha } \left( 0 \right)} \\ {x_{\beta } \left( 0 \right)} \\ \end{array} } \right] = (\bar{D} - \bar{S})^{{ - 1}} \left[ {c_{\alpha } {\text{ }}c_{\beta } } \right]\left[ {\begin{array}{*{20}c} { + 1} \\ { - 1} \\ \end{array} } \right]$$

According to Lemma 4^54^, each entry of (*D-S*)^*−*1^[*c*_*α*_* c*_*β*_] is nonnegative and each row sum of (*D-S*)^−1^[*c*_*α*_* c*_*β*_] is equal to 1. Therefore, the steady state of each normal agent is a convex combination of + 1 and − 1.

Further, we propose the measure of total support of normal nodes to node *α* (hereafter referred to as total support/impact of *α*) against perturbation from *β* as follows:

12 $$ToS(\alpha ) = \mathop \sum \limits_{{\gamma \in \user2{V}\backslash \{ {\mathbf{\alpha }}\} }} \bar{x}_{\gamma }$$

where $\bar{x}_{\gamma }$ is the steady value of node $\gamma$ if an undirected link is added between node *β* and $\gamma$. Total support of *α—ToS*(*α*)—is computed by Algorithm S1 (see in Supplementary file). Driver agent of the network is identified by $C = \mathop {\max }\limits_{{\alpha \in V}} ToS\left( \alpha \right)$. Driver agents may include a few nodes with the same total support, whose value may be only approximately computed because of network noise from the issues of measurement techniques and inherent natural variation^51^. Therefore, we selected driver agents from the top three nodes with the highest total support, which are also considered drug target genes in a disease network^32^.

### An illustration example

**Figure 2:**
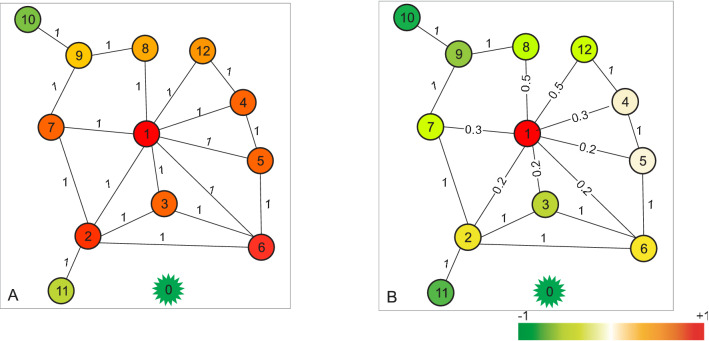
An illustrative example of how network structure influences the competitive impact results between driver gene and drug. A disease network with 12 genes and 19 interactions is given. Node 1 (red) is a driver gene whose state is fixed by 1. Node 0 (green) is a drug whose state is fixed by −1. An undirected interaction is temporarily added between the drug and each normal gene for computing support of the normal gene to the driver gene against impact from the drug. The state of each gene converges to a steady value which is a convex combination of the competitors’ states, and does not depend on the initial states of genes. The color gradient represents support bias to two competitors. (**A**) The weights are kept at the default value of 1 for all links. The result shows that most genes in the network impacted by the driver gene, except 10 and 11 (**B**) A handful of links are changed in weight. Interestingly, there are large fluctuations in the network. Most genes in the network turn to impacted by the drug.

Figure 2 shows outside competitive dynamics on two disease networks that have the same number of genes but different adjacency structures. We have considered a driver gene (node 1) and a drug (node 0) as two competitors in each network with fixed states *x*_1_ =  + 1 and *x*_0_ =  *− *1. To model the interaction between the drug and each normal gene, an undirected link was temporarily added between the drug and the normal gene, whenever this gene adjusts its state. We then computed the support of each gene to the driver gene against opposition impact from the drug. Stable states of normal genes were computed according to Eq. (6). A red (green) node represented a gene with a positive (negative) state. The darker the color, the larger was the absolute value of the state. White color nodes represented neutral agents. For network (A), the weights were maintained at the default value of 1 for all links. The result showed that most genes in the network, except 10 and 11, were impacted by the driver gene. For network (B), a handful of links were changed in weight. Most genes in the network converted to support the opponent drug outside the network. We observed that adjacency weights influenced the outside competition results, demonstrating the presence of large fluctuations in the network. In the following section, we will consider unweighted networks by considering that the weight of every link in a network is 1.

### Relationship between total support and hierarchical closeness

**Figure 3:**
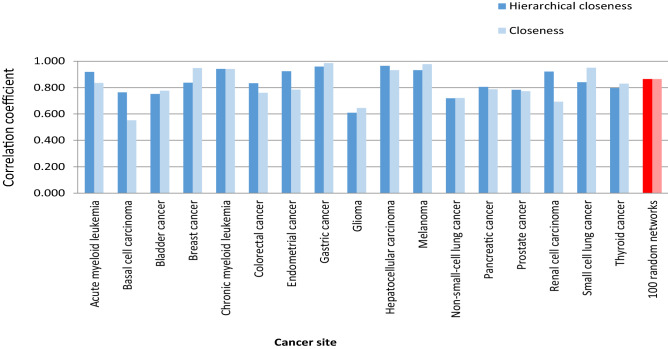
Correlation coefficient between total support and closeness, hierarchical closeness. Blue columns indicate the results on 17 cancer signaling networks and the red represents those on 100 random directed networks generated with |*V*| = 50 and 49 ≤ |*E*|≤ 100. Dark red represents the value of hierarchical closeness (R = 0.866; P = 0.0001) whereas light red is the value of closeness (R = 0.866; P = 0.0001) (see Table S1 for details).

**Figure 4:**
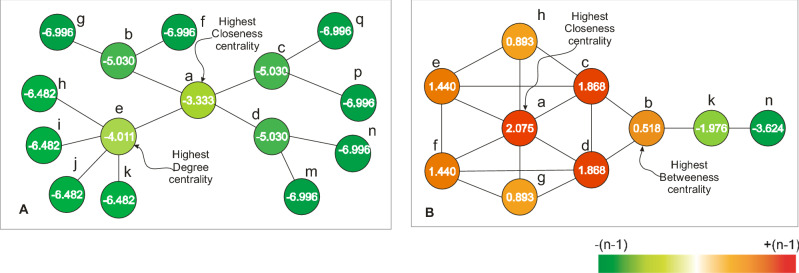
An illustrative example of the comparison between hierarchical closeness and other popular centralities for the prediction of total support. (**A**) Comparison with degree centrality. The highest closeness centrality outperforms the highest degree centrality in total support from the other nodes. (**B**) Comparison with betweenness centrality. The highest closeness centrality outperforms the highest betweenness centrality in total support from the remaining nodes. The color gradient represents the total support of a node ranging from −(n − 1) to + (n − 1), where *n* is the number of nodes. Note that hierarchical closeness and closeness exhibit the same ranking result in these two networks.

Closeness is one of the most well-known structural centrality measures^46^ wherein a node is defined as the inverse of the total sum of the shortest distance to the remaining nodes in an undirected network, and its effectiveness for disease gene prediction has frequently been reported for undirected biological networks^47,48,55,56^. Moreover, the closeness definition can be slightly modified to be properly used in a directed network^49^. In another study, Tran (2014) proposed an extensive closeness centrality called hierarchical closeness, which is a generalized measure of closeness centrality because it provides ranking results similar to that of closeness on an undirected network as well as functions efficiently on a directed or unconnected network^29^. The study found that hierarchical closeness outperforms other structural centrality measures in disease gene prediction. Moreover, the study showed that genes with a high level of hierarchical closeness were able to encode proteins in the extracellular matrix and receptor proteins in a human signal network. Particularly, hierarchical closeness was used to identify biomarker genes ^25^, which have also been reported as cancer therapeutic targets in cancer signaling networks^32^. Both findings suggest that hierarchical closeness denotes both biomarker genes and drug target genes in cancer signaling networks. Considering the above results, we examined the relationship between hierarchical closeness of a node and total support of the node. To this end, the Barabasi–Albert network growth model^57^ was used to generate random directed networks with the scale-free property inducing a few hub nodes and several non-hub nodes, as observed in real signaling networks. Investigation on the random networks generated by the model could statistically prove a network property in a generalized case. Experiment on 17 molecular signaling networks of cancers and 100 random directed networks generated with |*V*|= 50 and 49 ≤|*E*|≤ 100 showed that total support of each node positively correlates with both closeness and hierarchical closeness of the node (Fig. 3) because the correlation coefficients between total support and closeness (hierarchical closeness) on random networks was 0.866 (P < 0.05). To illustrate the performance of hierarchical closeness on the prediction of total support, we demonstrated that the closeness centrality of an existing node is a significantly better predictor of total support of the node than the degree/betweenness centrality (Fig. 4). This result further provided an important basis to suggest that total support can be predicted by hierarchical closeness which indicates the closeness of a node to all other nodes. To clarify the role of total support in the identification of cancer therapeutic targets, we conducted an extensive experiment, the findings from which are detailed in the following section.

### Identification of anticancer drug target genes by total support

Recent research on cancer signaling networks has shown that genes with high hierarchical closeness values were considered cancer biomarkers^25^, which also are often cancer therapeutic targets^32^. In the previous section, it was demonstrated that the hierarchical closeness of a node correlates with the total support of the node. These findings suggest that total support can accurately predict biomarker genes as well as drug target genes in a cancer signaling network. Considering these findings, we examined three genes with the highest total support of 17 cancer cell signaling networks (Table 1). Interestingly, 42 out of 51 genes (82%) were previously identified as anticancer drug target genes. For example, the three genes GRB2, FLT3, and PML, which were found in the acute myeloid leukemia (AML) signaling network, were considered key drug target genes. FLT3 is a common therapeutic target because it is frequently overexpressed or mutated, and its mutations indicate poor prognosis in AML. The development of FLT3 inhibitors leads to the recent approval of two drugs: Midostaurin (PKC412) and Gilteritinib (ASP2215) for the treatment of FLT3 mutant AML^58^. AML has a subtype called Acute promyelocytic leukemia (APL). The treatment of APL has dramatically been improved with the introduction of two synergism drugs of all-trans retinoic acid (ATRA) and arsenic trioxide (ATO) related to the targeting of genes PML^59^. The effect of GRB2 in AML treatment is under clinical trial with a phase II study of BP1001 that is a liposome-incorporated GRB2 antisense oligonucleotide for inhibition of GRB2 expression^60^. For another example, among the three genes HGF, MET, and EGLN2 identified in the renal cell carcinoma signaling network, HGF^61^ and MET^62,63^ have been known as potential and approved drug target genes, respectively; the third gene EGLN2 may be a promising anticancer drug target gene. A recent study demonstrated that the efficiency of cancer treatment may be significantly enhanced by combining drugs against multiple tumor specific drivers genes^64^. Taken together, 42 of the 51 genes (82%) that were reported as therapeutic targets confirmed that the top three genes with the highest total support score have the highest likelihood to be anticancer drug target genes; the 8 remaining genes that have not yet been completely investigated may be promising anticancer drug target genes. Although the predicted top three target genes of 8 networks don't have approved target genes, these predicted rank lists are also meaningful for further reference.

**﻿Table 1:** Anticancer drug target genes are identified by total support ranking. In the table, C1, C2, and C3 denote NCBI gene symbols of the top three genes with the highest total support. The underlined and bold genes (42 out of 51) were previously reported as anticancer drug target genes (see Table S2), in which genes in bold are approved for drug manufacture whereas the remaining ones are clinical trial/potential. The remaining neither underlined nor bold genes in the top three highest-ranking genes that have not yet been fully investigated may be promising anticancer drug target genes.

Cancer site	Network properties		Candidate anticancer drug target genes by Totalsupport		
	The number of nodes	The number of links	Cl	C2	C3
Acute myeloid leukemia	66	183	GRB2	**FLT3**	**PML**
Basal cell carcinoma	59	550	SUFU	**SMO**	GLI3
Bladder cancer	29	58	RASSF1	**FGFR3**	HRAS
Breast cancer	144	773	**LRP6**	LRP5	**WNT1**
Chronic myeloid leukemia	76	182	CRK	CRKL	GAB2
Colorectal cancer	74	195	**EGFR**	**GRB2**	KRAS
Endometrial cancer	51	117	EGF	EGFR	AXIN1
Gastric cancer	148	682	LRP6	LRP5	WNT7A
Glioma	74	310	CALM1	CALML5	CALM2
Hepatocellular carcinoma	167	769	LRP6	WNT3A	WNT7A
Melanoma	69	580	FGF2	FGF1	HGF
Non-small-cell lung cancer	65	157	**ALK**	EML4	KRAS
Pancreatic cancer	75	163	**KRAS**	AKT2	AKT1
Prostate cancer	84	375	**IGF-1**	INS	PDGFB
Renal cell carcinoma	56	154	HGF	**MET**	EGLN2
Small cell lung cancer	91	356	ITGB1	COL4A1	LAMB3
Thyroid cancer	37	84	NTRK1	TPR	TPM3

### Comparison with other methods

**Figure 5:**
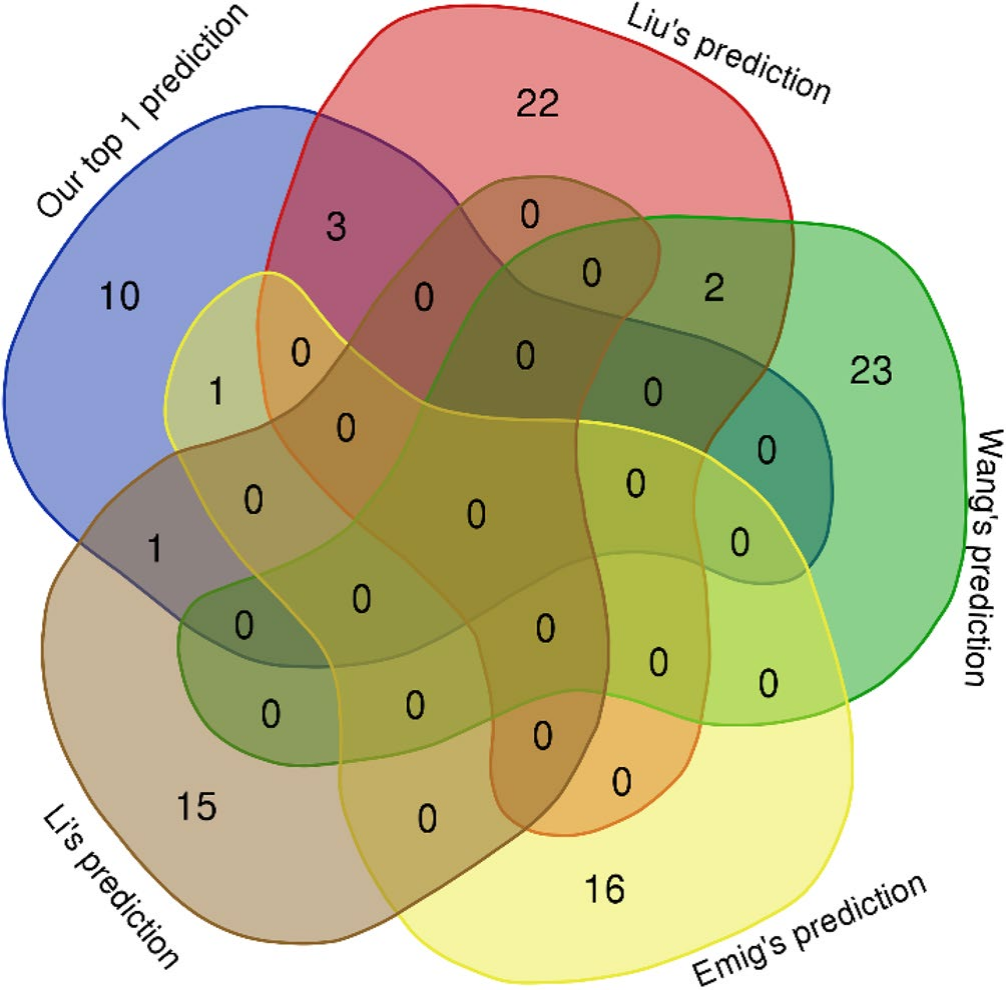
Comparison with other prediction results. The Venn diagram was drawn based on the intersection of the predicted anticancer drug target genes in four previous reports and our top 1 prediction. Our top 1 prediction shares consistency with the most predictions of different methods, i.e., 3 out of 4 predictions. The figure was drawn with the online tool http://bioinformatics.psb.ugent.be/webtools/Venn/.

We compared our method with four previous ones, namely: Liu's prediction^65^, Wang's prediction^66^, Emig's prediction^67^, and Li's prediction^68^. These methods are network-based approaches to predict drug targets of common cancers. Liu's prediction suggested 27 potential anticancer drug targets by a network-based screening of gene pairs on human cancer signaling network. Wang's prediction identified 25 candidate cancer drug targets by network score from genes sensitive with p53 mutation, which occurs in more than half of all human cancer cases. Emig's prediction found 17 cancer drug target genes by a combination of four network methods, namely: Neighborhood Scoring, Interconnectivity, Network Propagation, and Random Walks on a molecular interaction network associated with microarray experiment data. Li's prediction proposed 16 candidate anticancer drug targets using Random Walks on heterogeneous networks integrated from multi-source data. To compare with the above methods, we used our top 1 prediction (C1 in Table 1) including a list of 15 unique elements for common cancers. Note that the number of elements in our list was the smallest among the five predictions. We used top 1 prediction rather than top 3 prediction to guarantee that the comparison is not biased towards us because the size of our list is greater than those of the other lists. The Venn diagram in Fig. 5 showed that our's prediction and Liu's prediction had the same biggest number of intersection elements, i.e., 5 genes. Our intersection genes were composed of HGF, FGF2, ITGB1, EGFR, and GRB2, which most relates to growth factor. Especially, with the smallest number of elements, our prediction shared consistency with three different methods whereas Liu’s prediction agreed with only two methods (see Table S3). This implies that our prediction outperforms the others, for it shared overlap with most of the remaining predictions whereas the number of elements was the smallest. All these methods have been used separately for predicting anticancer drug targets, and we believed using them together will provide better results.

## Discussion

Knowledge discovery of anticancer drug target genes is key to the research and development of successful drugs for cancer treatment. In the present study, an outside competitive dynamics model was used on the complex network to identify the drug target genes by calculating the total support of each agent in the cancer signaling network. First, we proposed a novel dynamic model for outside competition in a complex network and defined the total support of a node in the model. Total support of a leader node indicates the support degree of the other nodes to the leader as well as, conversely, represents the impact of the leader on the remaining nodes against the outside impact. Therefore, nodes with the highest total support score act as driver nodes that easily navigate the state of the other nodes in the network via signaling to the neighbors from the drivers. Second, we showed an illustrative example of the working of the model in a disease network and the influence of adjacency weights on the outside competition results. This example demonstrates that the number of links as well as the quality of links decides the impact of a leader on other nodes. Third, by experimenting on 17 molecular signaling networks of cancers and 100 random directed networks, we showed that the total support of each node positively correlates with closeness and hierarchical closeness of the node. This finding indicates that the closer a node is to the remaining nodes, the more it receives support from them and the better it influences them. Note that a node with the highest closeness ranking is unnecessarily a network hub. Therefore, it suggests that a driver node often is at the central position of a disease network, where it may have only a few interactions to control the remaining nodes. Recent studies have reported that driver nodes often have the highest betweenness^69^ or the highest degree^70^ in some network types. We compared the prediction of total support between hierarchical closeness and degree/betweenness to demonstrate that a node with the highest closeness may be a better driver node than the others (Fig. 4). This finding was relatively different from that of the previous reports^69,70^. Note that we do not refute the previous findings because the closeness centrality used in the present study is a new version that was different from that used previously. Fourth, considering a finding that the top highest hierarchical closeness-ranking nodes often play biomarker genes^25^ and a report that backbone driver nodes often act as both biomarkers and drug target genes in the cancer signaling network^32^, we conducted an extensive investigation on the performance of total support in the identification of drug target genes. Of note, when we examined the top 3 genes with the highest total support, 82% of the genes were previously found to be anticancer drug target genes and the remaining genes that have not yet been completely investigated may be promising anticancer drug target genes. This result is consistent with that of a previous report^32^ and is evidence that genes with high support from the other genes in a cancer signaling network are most likely to be anticancer drug target genes and act as driver nodes in the network. Although the predicted top three target genes of 8 networks don't have approved target genes, these predicted rank lists are also meaningful for further reference. Finally, we validated our cancer drug target prediction by comparison with four previous network-based methods. As a result, our top 1 prediction shared the highest consistency among predictions by different methods. Overall, the outside competitive dynamics model contributes to the identification of both anticancer drug targets and driver agents in the cancer signaling network.

Our study showed that total support is a novel dynamic centrality measure for effective identification of anticancer drug target genes on cancer signaling networks. Although we only applied the outside competitive dynamics model on cancer signaling networks to identify anticancer drug target genes, the model can also be generalized to identify driver agents in a complex network. The positive relationship between total support and hierarchical closeness suggests that a driver agent in a network might use techniques such as hierarchical closeness optimization to adjust the network structure for winning the competition to the outside opponent competitor in various network types. However, obtaining valid evidence to demonstrate that the model functions satisfactorily in such a network type remains a key challenge. In addition, the algorithm to compute the total support presented in this study is limited by the long-running time required, particularly for large networks. All these issues will be considered in future studies.

## Supplementary Information

Supplementary Information.
