# Supplementary material for: Identification of anticancer drug target genes using an outside competitive dynamics model on cancer signaling networks
Source: Sci Rep. 2021 Jul 8;11:14095. doi: 10.1038/s41598-021-93336-z (PMC8266823; doi:10.1038/s41598-021-93336-z)
Supplement: Supplementary file 1 — Supplementary Information. [file 41598_2021_93336_MOESM1_ESM.docx]

| **Identification of** **anticancer drug target genes using an outside competitive dynamics model on cancer signaling networks** Tien-Dzung Tran^1,2,^* and Duc-Tinh Pham^1,3^ ^1^Complex Systems and Bioinformatics Lab, Hanoi University of Industry, 298 Cau Dien street, Bac Tu Liem District, Hanoi, Vietnam;  ^2^Department of Software Engineering, Faculty of Information and Communication Technology, Hanoi University of Industry, 298 Cau Dien street, Bac Tu Liem District, Hanoi, Vietnam.  ^3^Graduate University of Science and Technology, Vietnam Academy of Science and Technology, Hanoi, Vietnam  *Corresponding: [trantd@haui.edu.vn](mailto:trantd@haui.edu.vn) |
| --- |

Supplementary Information

| **Algorithm S1.** Computation of total support of nodes in a network *G*(*V*,*E*) to a node *α* against outside impact. Application of this algorithm in the identification of cancer drug target genes can be found at <https://github.com/tinhpd80/Total-support> | |
| --- | --- |
| 1 | **function** *[X_t_]* *InsideCompetition(G(V,E), Leaders ⊂ V, AgainstLeaders ⊂ V)* |
| 2 | *maxIterations*←*10000;* |
| 3 | *Epsilon ← 4.94e-324;* |
| 4 | *ε* ←*1/(Max*(total weights of out-links of *v*, ∀*v*∈*V) - Epsilon;* |
| 5 | *X_t_*←**new** *Dictionary<node,state>;* |
| 6 | *X_t+1_* ←**new** *Dictionary<node,state>;* |
| 7 | **for** (*Node* **in** *V* ) **do** |
| 8 | *X_t_[Node]* ←*0;* |
| 9 | **end for** |
| 10 | **for** (*Leader* **in** *Leaders*) **do** |
| 11 | *X_t_[Leader] ←1;* |
| 12 | *X_t+1_[Leader] ←1;* |
| 13 | **end for** |
| 14 | **for** (*AgainstLeader* **in** *AgainstLeaders*) **do** |
| 15 | *X_t_[AgainstLeader]* ← *−1;* |
| 16 | *X_t+1_[AgainstLeader]* ← *−1;* |
| 17 | **end for** |
| 18 | *Error ← 0;* |
| 19 | *t ← 0;* |
| 20 | **do** |
| 21 | *Error← 0;* |
| 22 | **for** (*u* **in** *V*) **do** |
| 23 | **if** *(Leaders* contain *u* **or** *AgainstLeaders* contain *u)* **continue*;*** |
| 24 | **end if** |
| 25 | *r←0;* |
| 26 | **for** (*v* **in** *Neighbors of u*) **do** |
| 27 | *r← r + weight(u,v)*(X_t_[v]- X_t_[u]);* |
| 28 | **end for** |
| 29 | *X_t+1_[u]* ← *X_t_[u]+ ε* r;* |
| 30 | *Error ← Error + Math.Abs(X_t_[u] - X_t+1_[u]);* |
| 31 | **end for** |
| 32 | *Temp←X_t_;* |
| 33 | *X_t_← X_t+1_;* |
| 34 | *X_t+1_←Temp;* |
| 35 | *t ← t +1;* |
| 36 | **while** *(Error > Epsilon && t < maxIterations);* |
| 37 | **return** *X_t_;* //Output as stable states of nodes as *t*→∞ |
| 38 | **end** |
| 39 | **function** *[Support]* *OutsideCompetition (G(V,E),* α *∈V)* |
| 40 | *Support←* **new** *Dictionary<node,state>;* |
| 41 | *β←* **new** Node |
| 42 | *NormalAgents← V \{β,* α*} ;* |
| 43 | **for** (*γ* **in** *NormalAgents* ) **do** |
| 44 | *e←* **new** *Edge(β, γ);* |
| 45 | *E= E ∪ {e};* |
| 46 | $\bar{X}$*← InsideCompetition(G(V,E), {*α*},{β});* |
| 47 | *Support[γ] ←* $\bar{X}$*[γ];* |
| 48 | *E= E \ {e};* |
| 49 | **end for** |
| 50 | **return** *Support;*//Support of nodes to α when connecting to *β* |
| 51 | **end** |
| 52 | **function**  [*TotalSupport*] *ToS(G(V,E),* α *∈ V)* |
| 53 | *Support←* **new** *Dictionary<node,state>;* |
| 54 | *Support← OutsideCompetition (G(V,E),* α*);* |
| 55 | *TotalSupport ← 0;* |
| 56 | **for** (*γ* **in** *V*\{α}) **do** |
| 57 | *TotalSupport ← TotalSupport + Support[γ]* |
| 58 | **end for** |
| 59 | **return** *TotalSupport*; //Total support of nodes to α |
| 60 | **End** |

| **Table S1**. Correlation coefficient (R) between total support and closeness/hierarchical closeness of 17 cancer types (all R have P < 0.0001) | | |
| --- | --- | --- |
| **Cancer name** | **R(total support, hierarchical closeness)** | **R(total support, closeness)** |
| Acute myeloid leukemia | 0.919 | 0.836 |
| Basal cell carcinoma | 0.764 | 0.552 |
| Bladder cancer | 0.752 | 0.776 |
| Breast cancer | 0.837 | 0.948 |
| Chronic myeloid leukemia | 0.942 | 0.941 |
| Colorectal cancer | 0.834 | 0.760 |
| Endometrial cancer | 0.924 | 0.784 |
| Gastric cancer | 0.959 | 0.986 |
| Glioma | 0.610 | 0.646 |
| Hepatocellular carcinoma | 0.965 | 0.932 |
| Melanoma | 0.932 | 0.978 |
| Non-small-cell lung cancer | 0.719 | 0.721 |
| Pancreatic cancer | 0.805 | 0.787 |
| Prostate cancer | 0.783 | 0.773 |
| Renal cell carcinoma | 0.922 | 0.693 |
| Small cell lung cancer | 0.841 | 0.951 |
| Thyroid cancer | 0.797 | 0.830 |
| 100 random directed networks generated with  \|V\| = 50 and 49 ≤ \|E\| ≤ 100 | 0.866  (P = 0.0001) | 0.866  (P = 0.0001) |

| **Table S2.**  Previously reported anticancer drug target genes among the top three genes with the highest total support. Genes in bold are approved for drug manufacture whereas the remaining ones are clinical trial/potential. | |
| --- | --- |
| **Cancer site** | **Anticancer drug target genes** |
| Acute myeloid leukemia | [GRB2](https://doi.org/10.1002/gcc.20344)[[^1^](#_ENREF_1)](#_ENREF_1); [**FLT3**](https://doi.org/10.2174/1381612043384394)[**^2^**](#_ENREF_2); **PML**[**^3^**](#_ENREF_3) |
| Basal cell carcinoma | SUFU[^4^](#_ENREF_4); **SMO**[**^5^**](#_ENREF_5); GLI3[^6^](#_ENREF_6) |
| Bladder cancer | RASSF1[^9^](#_ENREF_9) [**^10^**](#_ENREF_10)**^,^**[**^11^**](#_ENREF_11)**, FGFR3**[**^8^**](#_ENREF_8)**,** HRAS[^7^](#_ENREF_7) |
| Breast cancer | **[LRP6](https://www.pnas.org/content/107/11/5136.short)**^[12](#_ENREF_12)^; [LRP5](https://journals.plos.org/plosone/article?id=10.1371/journal.pone.0004243)[^13^](#_ENREF_13)^,^[^14^](#_ENREF_14); **[WNT](https://pubmed.ncbi.nlm.nih.gov/22846569/)1**^[15](#_ENREF_15)^ |
| Chronic myeloid leukemia | [CRK](https://dx.doi.org/10.3889%2Foamjms.2018.420)[^16^](#_ENREF_16); [CRKL](https://dx.doi.org/10.18632%2Foncotarget.2072)^[17](#_ENREF_17)^; [GAB2](https://www.nature.com/articles/leu2012222)^[18](#_ENREF_18)^ |
| Colorectal cancer | **[EGFR](https://doi.org/10.3978/j.issn.1000-9604.2014.01.10)**[^19^](#_ENREF_19)^,^[^20^](#_ENREF_20); **[GRB2](https://doi.org/10.1007/s00432-009-0574-8)**^[21](#_ENREF_21)^; [KRAS](https://www.ncbi.nlm.nih.gov/pmc/articles/PMC5850913/)^[22](#_ENREF_22)^ |
| Endometrial cancer | [EGF](https://molecular-cancer.biomedcentral.com/articles/10.1186/1476-4598-9-166)^[23](#_ENREF_23)^; [EGFR](https://molecular-cancer.biomedcentral.com/articles/10.1186/1476-4598-9-166)[^23^](#_ENREF_23)^,^[^24^](#_ENREF_24) |
| Gastric cancer | LRP6[^25^](#_ENREF_25); [LRP5](https://dx.doi.org/10.3978%2Fj.issn.1000-9604.2014.08.22)[^26^](#_ENREF_26); WNT7A[^27^](#_ENREF_27) |
| Glioma | [CALM1](https://doi.org/10.1109/HIBIT.2013.6661681)[^28^](#_ENREF_28) |
| Hepatocellular carcinoma | [LRP6](https://doi.org/10.1186/s12967-014-0259-1)[^30^](#_ENREF_30)^,^[^31^](#_ENREF_31), [WNT3A](https://www.spandidos-publications.com/10.3892/ijo.2017.4112)[^29^](#_ENREF_29) |
| Melanoma | FGF2[^36^](#_ENREF_36), FGF1[^35^](#_ENREF_35)^,^[^36^](#_ENREF_36), [HGF](https://www.nature.com/articles/modpathol2013226)[^32-34^](#_ENREF_32) |
| Non-small-cell lung cancer | **ALK**[^37^](#_ENREF_37)^,^[^38^](#_ENREF_38); EML4[^39^](#_ENREF_39), KRAS[^40-42^](#_ENREF_40) |
| Pancreatic cancer | [**KRAS**](http://doi.org/10.1158/1535-7163.MCT-11-0269)[^43^](#_ENREF_43)^,^[^44^](#_ENREF_44); [AKT2](https://doi.org/10.1152/ajprenal.00357.2012)[^45^](#_ENREF_45); AKT1[^46^](#_ENREF_46) |
| Prostate cancer | **IGF-1**[^47-49^](#_ENREF_47) |
| Renal cell carcinoma | [HGF](https://www.ncbi.nlm.nih.gov/pmc/articles/PMC3081175/)[^50^](#_ENREF_50); [**MET**](https://dx.doi.org/10.1200%2FJCO.2012.43.3383)[^51^](#_ENREF_51)^,^[^52^](#_ENREF_52) |
| Small cell lung cancer | ITGB1[^53^](#_ENREF_53); COL4A1[^54^](#_ENREF_54) |
| Thyroid cancer | [NTRK1](https://doi.org/10.1016/j.mce.2009.10.009)^[55-57](#_ENREF_55)^; [TPM3](https://www.nature.com/articles/6690920)[^56^](#_ENREF_56) |

| **Table S3.** Comparison between Our top 1 prediction and four previous network-based predictions. | | |
| --- | --- | --- |
| **Input**: | | |
| **List names** | **Number of elements** | **Number of unique elements** |
| Our top 1 prediction | 15 | 15 |
| Liu's prediction | 27 | 27 |
| Emig's prediction | 17 | 17 |
| Li's prediction | 16 | 16 |
| Wang's prediction | 25 | 25 |
| **Overall number of unique elements** | | **93** |
| **Output:** | | |
| **Name** | **Total** | **Elements** |
| Liu's prediction Our top 1 prediction | 3 | HGF FGF2 ITGB1 |
| Emig's prediction Our top 1 prediction | 1 | EGFR |
| Li's prediction Our top 1 prediction | 1 | GRB2 |
| Liu's prediction Wang's prediction | 2 | CDK1 PLK1 |
| Our top 1 prediction | 10 | KRAS SUFU NTRK1 RASSF1 EGF LRP6 ALK CRK IGF-1 CALM1 |
| Liu's prediction | 22 | LYN YES1 BID IGF1 XIAP APAF1 IL6 PTGS2 TH PRKAB1 TNF HDAC1 NFKB1 WEE1 CD4 PRKCZ CDK2 CDC42 CDK5 PPARA CASP3 E2F1 |
| Wang's prediction | 23 | CHKA BUB1 MTOR PDGFRA PRKCSH CHEK2 CDK16 MARK2 STK17B NEK2 CSNK2B RAF1 CDC7 AURKA AK2 CERK BRD2 STK38 PLK4 SRPK1 UCK2 TTK PKC |
| Emig's prediction | 16 | c-Fos NF-kB p50/p65 AP-1 STAT3 GCR-alpha HIF1A RelA (p65 NF-kB subunit) c-Jun STAT5 STAT5B p53 c-Src STAT5A CDK1 (p34) NF-kB JAK1 |
| Li's prediction | 15 | TRAF2 NOTCH2 NL N2N MTUS2 ASH KRT40 LAPSER1 SIRT1 KA36 GOLGA2 NCK1 KRT31 KIAA1813 LZTS2 NCK |

References

1 Ohanian, M. *et al.* A phase II study of BP1001 (liposomal Grb2 antisense oligonucleotide) in patients with hematologic malignancies. *Journal of Clinical Oncology* **38**, TPS7561-TPS7561, doi:10.1200/JCO.2020.38.15_suppl.TPS7561 (2020).

2 Gebru, M. T. & Wang, H.-G. Therapeutic targeting of FLT3 and associated drug resistance in acute myeloid leukemia. *Journal of Hematology & Oncology* **13**, 155, doi:10.1186/s13045-020-00992-1 (2020).

3 Testa, U. & Lo-Coco, F. Targeting of leukemia-initiating cells in acute promyelocytic leukemia. *Stem cell investigation* **2**, 8-8, doi:10.3978/j.issn.2306-9759.2015.04.03 (2015).

4 Ogden, T., Higgins, S., Elbaum, D. & Wysong, A. The relevance of a suppressor of fused (SUFU) mutation in the diagnosis and treatment of Gorlin syndrome. *JAAD case reports* **4**, 196-199, doi:10.1016/j.jdcr.2017.10.011 (2018).

5 (!!! INVALID CITATION !!! 5).

6 Wang, B., Fallon, J. F. & Beachy, P. A. Hedgehog-Regulated Processing of Gli3 Produces an Anterior/Posterior Repressor Gradient in the Developing Vertebrate Limb. *Cell* **100**, 423-434, doi:10.1016/S0092-8674(00)80678-9 (2000).

7 Sugita, S. *et al.* HRAS as a potential therapeutic target of salirasib RAS inhibitor in bladder cancer. *Int J Oncol* **53**, 725-736, doi:10.3892/ijo.2018.4435 (2018).

8 Casadei, C. *et al.* Targeted therapies for advanced bladder cancer: new strategies with FGFR inhibitors. *Therapeutic advances in medical oncology* **11**, 1758835919890285, doi:10.1177/1758835919890285 (2019).

9 Khandelwal, M. *et al.* RASSF1A-Hippo pathway link in patients with urothelial carcinoma of bladder: plausible therapeutic target. *Molecular and cellular biochemistry* **464**, 51-63, doi:10.1007/s11010-019-03648-y (2020).

10 Khandelwal, M. *et al.* Decitabine augments cytotoxicity of cisplatin and doxorubicin to bladder cancer cells by activating hippo pathway through RASSF1A. *Molecular and cellular biochemistry* **446**, 105-114, doi:10.1007/s11010-018-3278-z (2018).

11 Sun, X., Li, H., Sun, M., Yuan, Y. & Sun, L. Circulating tumor DNA RASSF1 methylation for predicting cancer risk: a diagnostic meta-analysis. *Future oncology (London, England)* **15**, 3513-3525, doi:10.2217/fon-2019-0219 (2019).

12 Zhou, L. *et al.* CDDO-Me Elicits Anti–Breast Cancer Activity by Targeting LRP6 and FZD7 Receptor Complex. *Journal of Pharmacology and Experimental Therapeutics* **373**, 149-159, doi:10.1124/jpet.119.263434 (2020).

13 Maubant, S. *et al.* LRP5 regulates the expression of STK40, a new potential target in triple-negative breast cancers. *Oncotarget* **9**, 22586-22604, doi:10.18632/oncotarget.25187 (2018).

14 Miller-Kleinhenz, J. *et al.* Dual-targeting Wnt and uPA receptors using peptide conjugated ultra-small nanoparticle drug carriers inhibited cancer stem-cell phenotype in chemo-resistant breast cancer. *Biomaterials* **152**, 47-62, doi:<https://doi.org/10.1016/j.biomaterials.2017.10.035> (2018).

15 Wu, D. *et al.* miR-140-5p inhibits the proliferation and enhances the efficacy of doxorubicin to breast cancer stem cells by targeting Wnt1. *Cancer Gene Therapy* **26**, 74-82, doi:10.1038/s41417-018-0035-0 (2019).

16 Hossain, S., Dubielecka, P. M., Sikorski, A. F., Birge, R. B. & Kotula, L. Crk and ABI1: binary molecular switches that regulate abl tyrosine kinase and signaling to the cytoskeleton. *Genes Cancer* **3**, 402-413, doi:10.1177/1947601912460051 (2012).

17 Frietsch, J. J. *et al.* LASP1 is a novel BCR-ABL substrate and a phosphorylation-dependent binding partner of CRKL in chronic myeloid leukemia. *Oncotarget* **5** (2014).

18 Wöhrle, F. U. *et al.* Gab2 signaling in chronic myeloid leukemia cells confers resistance to multiple Bcr-Abl inhibitors. *Leukemia* **27**, 118-129, doi:10.1038/leu.2012.222 (2013).

19 Miyamoto, Y., Suyama, K. & Baba, H. Recent Advances in Targeting the EGFR Signaling Pathway for the Treatment of Metastatic Colorectal Cancer. *International Journal of Molecular Sciences* **18**, 752 (2017).

20 Zhao, B. *et al.* Mechanisms of resistance to anti-EGFR therapy in colorectal cancer. *Oncotarget* **8**, 3980-4000, doi:10.18632/oncotarget.14012 (2017).

21 Ding, C. *et al.* The PEAK1–PPP1R12B axis inhibits tumor growth and metastasis by regulating Grb2/PI3K/Akt signalling in colorectal cancer. *Cancer Letters* **442**, 383-395, doi:<https://doi.org/10.1016/j.canlet.2018.11.014> (2019).

22 Porru, M., Pompili, L., Caruso, C., Biroccio, A. & Leonetti, C. Targeting KRAS in metastatic colorectal cancer: current strategies and emerging opportunities. *Journal of experimental & clinical cancer research : CR* **37**, 57-57, doi:10.1186/s13046-018-0719-1 (2018).

23 Nishimura, T. *et al.* Effect of the molecular targeted drug, erlotinib, against endometrial cancer expressing high levels of epidermal growth factor receptor. *BMC Cancer* **15**, 957, doi:10.1186/s12885-015-1975-5 (2015).

24 Albitar, L. *et al.* EGFR isoforms and gene regulation in human endometrial cancer cells. *Molecular Cancer* **9**, 166, doi:10.1186/1476-4598-9-166 (2010).

25 Zhang, G. *et al.* MicroRNA-610 inhibits tumor growth of melanoma by targeting LRP6. *Oncotarget* **8** (2017).

26 Liu, X. *et al.* LRP5 polymorphism-A potential predictor of the clinical outcome in advanced gastric cancer patients treated with EOF regimen. *Chin J Cancer Res* **26**, 478-485, doi:10.3978/j.issn.1000-9604.2014.08.22 (2014).

27 Wang, L., Wang, X. & Jiang, X. miR-127 suppresses gastric cancer cell migration and invasion via targeting Wnt7a. *Oncol Lett* **17**, 3219-3226, doi:10.3892/ol.2019.9955 (2019).

28 Özşık, O., Bakır-Güngör, B., Diri, B. & Sezerman, O. U. in *2013 8th International Symposium on Health Informatics and Bioinformatics.* 1-6.

29 Lu, C. *et al.* Expression of Wnt3a in hepatocellular carcinoma and its effects on cell cycle and metastasis. *Int J Oncol* **51**, 1135-1145, doi:10.3892/ijo.2017.4112 (2017).

30 Xiao, J. *et al.* Garlic-derived compound S-allylmercaptocysteine inhibits hepatocarcinogenesis through targeting LRP6/Wnt pathway. *Acta Pharmaceutica Sinica B* **8**, 575-586, doi:<https://doi.org/10.1016/j.apsb.2017.10.003> (2018).

31 Xiong, H. *et al.* GRP78 activates the Wnt/HOXB9 pathway to promote invasion and metastasis of hepatocellular carcinoma by chaperoning LRP6. *Experimental Cell Research* **383**, 111493, doi:<https://doi.org/10.1016/j.yexcr.2019.07.006> (2019).

32 Lezcano, C. *et al.* Evaluation of stromal HGF immunoreactivity as a biomarker for melanoma response to RAF inhibitors. *Modern Pathology* **27**, 1193-1202, doi:10.1038/modpathol.2013.226 (2014).

33 Cheng, H. *et al.* Co-targeting HGF/cMET Signaling with MEK Inhibitors in Metastatic Uveal Melanoma. *Molecular Cancer Therapeutics* **16**, 516-528, doi:10.1158/1535-7163.mct-16-0552 (2017).

34 Demkova, L. & Kucerova, L. Role of the HGF/c-MET tyrosine kinase inhibitors in metastasic melanoma. *Molecular Cancer* **17**, 26, doi:10.1186/s12943-018-0795-z (2018).

35 Eigner, K. *et al.* The unfolded protein response impacts melanoma progression by enhancing FGF expression and can be antagonized by a chemical chaperone. *Scientific Reports* **7**, 17498, doi:10.1038/s41598-017-17888-9 (2017).

36 Rezzola, S. *et al.* The Autocrine FGF/FGFR System in both Skin and Uveal Melanoma: FGF Trapping as a Possible Therapeutic Approach. *Cancers* **11**, 1305 (2019).

37 Golding, B., Luu, A., Jones, R. & Viloria-Petit, A. M. The function and therapeutic targeting of anaplastic lymphoma kinase (ALK) in non-small cell lung cancer (NSCLC). *Molecular Cancer* **17**, 52, doi:10.1186/s12943-018-0810-4 (2018).

38 Sgambato, A., Casaluce, F., Maione, P. & Gridelli, C. Targeted therapies in non-small cell lung cancer: a focus on ALK/ROS1 tyrosine kinase inhibitors. *Expert Rev Anticancer Ther* **18**, 71-80, doi:10.1080/14737140.2018.1412260 (2018).

39 Dhawan, A. *et al.* Collateral sensitivity networks reveal evolutionary instability and novel treatment strategies in ALK mutated non-small cell lung cancer. *Sci Rep* **7**, 1232, doi:10.1038/s41598-017-00791-8 (2017).

40 Román, M. *et al.* KRAS oncogene in non-small cell lung cancer: clinical perspectives on the treatment of an old target. *Molecular Cancer* **17**, 33, doi:10.1186/s12943-018-0789-x (2018).

41 Salgia, R., Pharaon, R., Mambetsariev, I., Nam, A. & Sattler, M. The improbable targeted therapy: KRAS as an emerging target in non-small cell lung cancer (NSCLC). *Cell Reports Medicine* **2**, 100186, doi:<https://doi.org/10.1016/j.xcrm.2020.100186> (2021).

42 Ferrer, I. *et al.* KRAS-Mutant non-small cell lung cancer: From biology to therapy. *Lung Cancer* **124**, 53-64, doi:<https://doi.org/10.1016/j.lungcan.2018.07.013> (2018).

43 Kim, S. T. *et al.* Impact of <em>KRAS</em> Mutations on Clinical Outcomes in Pancreatic Cancer Patients Treated with First-line Gemcitabine-Based Chemotherapy. *Molecular Cancer Therapeutics* **10**, 1993-1999, doi:10.1158/1535-7163.mct-11-0269 (2011).

44 Waters, A. M. & Der, C. J. KRAS: The Critical Driver and Therapeutic Target for Pancreatic Cancer. *Cold Spring Harb Perspect Med* **8**, doi:10.1101/cshperspect.a031435 (2018).

45 Sun, Y. *et al.* MiRNA-615-5p Functions as a Tumor Suppressor in Pancreatic Ductal Adenocarcinoma by Targeting AKT2. *PLOS ONE* **10**, e0119783, doi:10.1371/journal.pone.0119783 (2015).

46 Xu, R.-l. *et al.* Primate-specific miRNA-637 inhibited tumorigenesis in human pancreatic ductal adenocarcinoma cells by suppressing Akt1 expression. *Experimental Cell Research* **363**, 310-314, doi:<https://doi.org/10.1016/j.yexcr.2018.01.026> (2018).

47 Wang, S. *et al.* Circulating IGF-1 promotes prostate adenocarcinoma via FOXO3A/BIM signaling in a double-transgenic mouse model. *Oncogene* **38**, 6338-6353, doi:10.1038/s41388-019-0880-9 (2019).

48 Weyer-Czernilofsky, U. *et al.* Antitumor Activity of the IGF-1/IGF-2–Neutralizing Antibody Xentuzumab (BI 836845) in Combination with Enzalutamide in Prostate Cancer Models. *Molecular Cancer Therapeutics* **19**, 1059-1069, doi:10.1158/1535-7163.mct-19-0378 (2020).

49 Hussain, S. A. *et al.* Targeting IGF-1/2 with xentuzumab (Xe) plus enzalutamide (En) in metastatic castration-resistant prostate cancer (mCRPC) after progression on docetaxel chemotherapy (DCt) and abiraterone (Abi): Randomized phase II trial results. *Journal of Clinical Oncology* **37**, 5030-5030, doi:10.1200/JCO.2019.37.15_suppl.5030 (2019).

50 Giubellino, A., Linehan, W. M. & Bottaro, D. P. Targeting the Met signaling pathway in renal cancer. *Expert review of anticancer therapy* **9**, 785-793, doi:10.1586/era.09.43 (2009).

51 Nandagopal, L., Sonpavde, G. P. & Agarwal, N. Investigational MET inhibitors to treat Renal cell carcinoma. *Expert Opinion on Investigational Drugs* **28**, 851-860, doi:10.1080/13543784.2019.1673366 (2019).

52 Alonso-Gordoa, T. *et al.* Targeting Tyrosine kinases in Renal Cell Carcinoma: “New Bullets against Old Guys”. *International Journal of Molecular Sciences* **20**, 1901 (2019).

53 Wang, X.-M. *et al.* Integrative Analyses Identify Osteopontin, LAMB3 and ITGB1 as Critical Pro-Metastatic Genes for Lung Cancer. *PLOS ONE* **8**, e55714, doi:10.1371/journal.pone.0055714 (2013).

54 Zhou, C., Chen, H., Han, L., Wang, A. & Chen, L. A. Identification of featured biomarkers in different types of lung cancer with DNA microarray. *Molecular biology reports* **41**, 6357-6363, doi:10.1007/s11033-014-3515-9 (2014).

55 Greco, A., Miranda, C. & Pierotti, M. A. Rearrangements of NTRK1 gene in papillary thyroid carcinoma. *Molecular and Cellular Endocrinology* **321**, 44-49, doi:<https://doi.org/10.1016/j.mce.2009.10.009> (2010).

56 Bounacer, A. *et al.* Search for NTRK1 proto-oncogene rearrangements in human thyroid tumours originated after therapeutic radiation. *British Journal of Cancer* **82**, 308-314, doi:10.1054/bjoc.1999.0920 (2000).

57 Tirrò, E. *et al.* Molecular Alterations in Thyroid Cancer: From Bench to Clinical Practice. *Genes* **10**, 709 (2019).
